# Comorbid ADHD and schizophrenia and the use of psychostimulants: a scoping review protocol

**DOI:** 10.1136/bmjopen-2024-090290

**Published:** 2024-10-23

**Authors:** Jordan Bamford, I Qurashi, Ariana Axiaq, Steven Marwaha, Nusrat Husain

**Affiliations:** 1 The University of Manchester Division of Psychology and Mental Health, Manchester, UK; 2 Institute of Population Health, University of Liverpool, Liverpool, UK, Liverpool, UK; 3 Queen's University Belfast Faculty of Medicine Health and Life Sciences, Belfast, UK; 4 University of Birmingham College of Life and Environmental Sciences, Birmingham, Birmingham, UK; 5 School of Medicine, The University of Manchester, Manchester, UK

**Keywords:** Mental Health, Schizophrenia and psychotic disorders, Clinical Pharmcology

## Abstract

**Abstract:**

**Introduction:**

Schizophrenia and attention deficit hyperactivity disorder (ADHD) are psychiatric disorders that have a profound impact on patients and healthcare systems globally. There is preliminary evidence suggesting a potential association between the two in terms of symptomatology and genetic underpinning. There is a paucity of guidance regarding pharmacological approaches for patients with comorbid ADHD and schizophrenia. There is a concern that psychostimulants may be more harmful than therapeutic. This scoping review protocol aims to systematically review the evidence for potential harm and benefit of psychostimulants among patients with comorbid ADHD and schizophrenia and identify research gaps.

**Methods and analysis:**

This scoping review will employ a systematic and iterative approach to identify and synthesise the literature on the topic of psychostimulant use among patients with comorbid schizophrenia and ADHD, based on Arksey and O’Malley’s framework. A search will be conducted in relevant databases, including MEDLINE (Ovid), Embase (Ovid), PsycINFO and ISI Web of Science. Additionally, grey literature will be sought. The scoping review will involve two independent reviewers screening the search results. The initial screen will be of title and abstract, and the subsequent full-text review will determine eligibility. A descriptive overview of the eligible studies will be provided. This scoping review has been registered at https://osf.io/cmn5s.

**Ethics and dissemination:**

There is a paucity of high-quality evidence available to clinicians when making decisions regarding the prescription of psychostimulants to patients with comorbid schizophrenia and ADHD. To the best of our knowledge, this will be the first scoping review to examine the evidence addressing this clinical scenario. This review, therefore, has the potential to contribute to decision-making processes for this patient group, thereby improving patient outcomes. Furthermore, as this review is designed to identify research gaps, we aim to contribute to the development of a research agenda that will benefit patients, clinicians and healthcare systems. The dissemination strategy will involve open access peer review publication and scientific presentations.

**STRENGTHS AND LIMITATIONS OF THIS STUDY**

- The scoping review protocol has been developed in accordance with the Preferred Reporting Items for Systematic Reviews and Meta-Analyses extension for Scoping Review guidelines.
- The data to be considered will be limited to those articles written in the English Language.
- The scoping review has been registered with the Open Science Framework in order to enhance transparency: https://osf.io/cmn5s.

## Introduction

### Schizophrenia and attention deficit hyperactivity disorder

 There is growing evidence of an association between schizophrenia and attention deficit hyperactivity disorder (ADHD).^1^ Schizophrenia is in many cases a lifelong mental illness characterised by distinct symptom clusters: ‘positive symptoms’, such as delusions, hallucinations and thought disorder; ‘negative symptoms’, such as flattened affect, amotivation, and anergy, and cognitive symptoms.^2^ It is associated with significant disability^3^ and premature mortality,^4^ with a substantial economic burden on healthcare systems worldwide.^5 6^ The lifetime prevalence of schizophrenia is approximately 1%,^7^ with peak incidence in the 20–29-year age group.^8^ The pathogenesis of schizophrenia is thought to begin in early neurodevelopment, owing to as a combination of genetic and early environmental risk factors.^9^ Epidemiological, serological and neuroimaging evidence implicates increased inflammation in the aetiology of schizophrenia.^10^

ADHD is a neurodevelopmental syndrome, characterised by inattentiveness, hyperactivity and increased impulsivity that is not commensurate with the stage of development; it is the most common psychiatric disorder in childhood.^11 12^ Globally, approximately 5% of children and 2.58% of adults are affected by ADHD.^11 13^ Those with ADHD compared with healthy controls have significantly disrupted sleep.^14^ The aetiology of ADHD is complex, with interaction between genetic factors and the environment. There is significant clinical heterogeneity due to comorbidity, differential impact on psychosocial functioning and gender effects.^15^ Iron deficiency has also been found to be associated with the severity of symptoms in ADHD.^16^ There is some evidence that those with ADHD demonstrate dysfunction of the fronto-subcortical region of the brain, which is rich in dopamine^17^; however, only modest structural differences have been identified when comparing those with ADHD to those without.^18^

### Comorbidity, shared genetics and commonalities

There is evidence to suggest an association between schizophrenia and ADHD. A study examining a cohort of patients presenting with first-episode psychosis requiring hospitalisation identified that 8.1% of this cohort had ADHD.^19^ Another study, which followed 208 young people, found that the relative risk of schizophrenia in patients who had a diagnosis of ADHD (compared with the general population) was 4.3 (95% CI 1.9 to 8.57).^20^ A systematic review has demonstrated that childhood ADHD is associated with an increased risk of subsequent psychosis.^21^ In England, a study involving 7403 participants found that ADHD symptomatology predicted psychosis, paranoid and auditory hallucinations.^22^ Furthermore, a large cohort study examining patients with ADHD, which included over 40 000 participants, identified that men with ADHD were at greater risk of having comorbid schizophrenia.^23^

The relationship between comorbid ADHD and schizophrenia may partly be explained by insights into the shared genetics of these diseases, leading to increased risk of developing both schizophrenia and ADHD.^24^ Other studies have reported on an association between psychosis symptomatology and genetic liability for ADHD, with the highest decile of ADHD genetic risk found to have an almost 2.5-fold increased likelihood of being in a psychosis spectrum cohort.^25^ Furthermore, genetic evidence indicates that ADHD is positively associated with treatment-resistant schizophrenia.^26^ In a large Swedish study comprising over 60 000 participants with ADHD, the risk of developing schizophrenia in first-degree relatives was found to be two times greater than in the control group.^27^

The comorbidity of schizophrenia and ADHD is further complicated by the fact that both conditions share commonalities of presentation, including inattention,^28^ deficits in executive function^29^ and reduced capacity for emotional processing,^30 31^ with both demonstrating problems with impulsivity, low frustration tolerance and impairment in social functioning.^32^ Preliminary evidence suggests that among those patients with schizophrenia who have comorbid ADHD, there is an increased prevalence of suicidal behaviour compared with those patients with just schizophrenia.^33^ Many of the common symptoms are also in part possibly explained by underlying sleep disturbance which is common in both conditions.^14 34^

### Clinical challenge

Pharmacological therapy for ADHD using psychostimulants is a first-line treatment in many countries.^35^ The use of ADHD medication has increased greatly for both children and adults.^36^ However, there are currently no specific guidelines for pharmacological approaches in patients with comorbid schizophrenia and ADHD. There is a general reluctance to use psychostimulants in comorbid schizophrenia and ADHD,^37^ as clinicians are concerned about inducing or aggravating psychotic symptoms. Early studies examining the impact of psychostimulants on patients with schizophrenia appeared to corroborate this and provided some evidence of worsening psychotic symptoms.^38^ A more recent review of patients with ADHD and early psychosis found that stimulant use may precipitate psychotic symptoms.^39^ It has been postulated that the administration of psychostimulants to patients with schizophrenia and comorbid ADHD may mitigate the effects of antipsychotics. However, this is not well researched, and it has been suggested that these drugs may not antagonise each other and may instead act in a synergistic manner.^32 40 41^

Previous reviews on psychostimulants, ADHD and schizophrenia have tended to focus on individual disorders. Some reviews have examined the risk of psychosis in patients prescribed psychostimulants for ADHD. Observational studies have found that psychostimulants have little effect on psychosis risk,^42^ and systematic reviews of randomised controlled trials of stimulant use for ADHD in children and adolescents were unable to determine if stimulants increase the risk of psychosis.^43^ Other reviews have considered the use of psychostimulants among patients with schizophrenia, finding some tentative evidence that negative symptoms may be improved without precipitating worsened positive symptoms.^44^ However, no reviews have yet been conducted on the use of psychostimulants among patients with comorbid schizophrenia and ADHD nor on the potential harms or benefits of such use.

## Objective

This scoping review will systematically explore evidence for the use of psychostimulants among patients who have comorbid schizophrenia and ADHD. This review will consider the therapeutic benefit, and any harm, of prescribing these medications among this cohort, with the aim of providing clarity for clinicians when making decisions regarding the prescription of therapeutic agents in patients with schizophrenia and ADHD. This protocol has been registered with the Open Science Framework: https://osf.io/cmn5s.

## Methods and analysis

This scoping review will use a systematic and iterative approach to identify and synthesise the literature on the topic of psychostimulant use for patients with comorbid schizophrenia and ADHD.^45^ This review will follow the methodological framework developed by Arksey and O’Malley^46^ and will take into account best practices for scoping reviews.^47 48^ This protocol is presented in the same approach as directed by the Preferred Reporting Items for Systematic Reviews and Meta-Analyses extension for Scoping Review guidelines.^48^ This scoping review protocol will detail the following stages^1^: identification of the research question^2^; identification of relevant studies^3^; selection of eligible studies^4^; charting the data; and^5^ collating, summarising and reporting of the results.

### Stage 1: identification of the research question

The overall research questions are as follows:

1. What is the existing evidence (of harm or benefit) for prescribing psychostimulants to patients who have comorbid schizophrenia and ADHD?
2. What is the existing evidence for prescribing psychostimulants to patients who have comorbid schizophrenia and ADHD and are also prescribed antipsychotics?
3. What are the existing gaps in the research for prescribing psychostimulants to patients with comorbid schizophrenia and ADHD?

### Stage 2: identification of relevant studies

This scoping review intends to use the Population, Concept and Context (PCC) framework developed by the Joanna Briggs Institute.^49^ The search strategy will be based on the framework presented in table 1.

**Table 1:** PCC framework of the scoping review

Criteria		
	Main concept	Alternate keywords
Population	Schizophrenia	Psychotic disorder OR Psychosis OR dementia praecox OR Schizophrenic psychosis OR schizophrenic disorder
	ADHD	Attention Deficit Disorder with Hyperactivity OR ADHD OR Attention Deficit Hyperactivity Disorder OR Attention Deficit and Hyperactivity Disorder OR Attention Deficit Disorder OR Hyperkinetic Disorder OR hkd OR Hyperkinetic syndrome
Concept	Psychostimulants	central nervous system stimulants OR psychostimulants OR stimulants OR methylphenidate OR amphetamine
Context	There will be no restrictions regarding the context of the studies. All geographic locations, cultural/subcultural, sociodemographic or economic contexts will be included	NA

ADHDattention deficit hyperactivity disorderPCCPopulation, Concept and Context

The search for empirical sources will be conducted in the following databases: MEDLINE (Ovid), Embase (Ovid), PsycINFO and ISI Web of Science. An initial search strategy for MEDLINE (Ovid) based on the PCC framework is presented in table 2. The presented search strategy will be adapted for each database. Only articles published in English will be included, with the focus on those published from the year 2000 onwards. In addition, the databases OpenGrey and Google Scholar will be searched for grey literature.

**Table 2:** MEDLINE (Ovid) search strategy

Search termline number	Conceptual term of interest	Search term entered	Number of hits
1	Schizophrenia	exp Schizophrenia	116 425
2	Psychotic Disorder	Exp psychotic disorders	59 431
3	Psychosis	Psychosis.mp	47 665
4	Dementia praecox	Dementia praecox.mp	580
5	Schizophrenia psychosis	Schizophrenic psychosis.mp	162
6	Schizophrenia disorder	Schizophrenia disorder.mp	87
7		1 or 2 or 3 or 4 or 5 or 6	178 453
8	Attention Deficit Disorder with hyperactivity	Exp Attention Deficit Disorder with Hyperactivity	35 449
9	ADHD	ADHD.mp	33 191
10	Attention Deficit Hyperactivity Disorder	Attention Deficit Hyperactivity Disorder.mp	32 357
11	Attention Deficit and Hyperactivity Disorder	Attention Deficit and Hyperactivity Disorder.mp	32 925
12	Attention Deficit Disorder	Attention Deficit Disorder.mp	36 337
13	HyperkineticDisorder	HyperkineticDisorder.mp	278
14	Hkd	Hkd.mp	179
15	Hyperkinetic syndrome	Hyperkinetic syndrome.mp	306
16		8 or 9 or 10 or 11 or 12 or 13 or 14 or 15	50 217
17	Psychostimulants	Exp central nervous system stimulants	103 837
18	Psychostimulant	Psychostimulant*.mp	7318
19	Stimulants	Stimulant*.mp	47 716
20	Methylphenidate	Exp methylphenidate	7898
21	Amphetamine	Exp amphetamine	19 707
22		17 or 18 or 19 or 20 or 21	128 991
23		7 and 16 and 22	158

ADHDattention deficit hyperactivity disorder

Completed 29 January 2024.

### Stage 3: study selection

The population in this scoping review will include adults over the age of 18, with diagnoses (based on Statistical Manual of Mental Disorders or International Classification of Diseases criteria) of ADHD and schizophrenia. The core concept being examined by this scoping review is the use of psychostimulants in this population. As detailed in table 1, the study will not be restricted to geographic or cultural contexts. We will not restrict by study design.

All papers identified through the search will be uploaded to EndNote, and duplicates will be removed. The scoping review will employ a two-stage review process to assess the suitability of studies for inclusion. The eligibility criteria have been generated and are detailed below. Two authors (JB and AA) will apply the eligibility criteria during the title/abstract review. Following each review stage, the agreement of the reviewers will be evaluated. In the event of a discrepancy, a third review (IQ/SM) will be consulted until a consensus is reached.

The eligibility requirements in relation to title/abstract are as follows:

1. Refers to patients with comorbid ADHD and schizophrenia
2. Refers to the use of psychostimulants
3. Published since 1 January 2000, in English
4. Any study design.

The full text of those articles that meet the title and abstract requirements will be obtained. These full-text articles will then be subjected to further screening. The full texts will be included in the scoping review if they meet the following criteria:

1. Definitions and diagnostic approaches of ADHD and schizophrenia are explicitly provided.
2. Population described, should only focus on those aged 18 or over
3. Use of psychostimulants described
4. Patient outcomes reported (harm or benefit).

### Stage 4: charting the data

The precise information extracted from the studies will be developed iteratively. A sample of the studies will be selected to determine and gain consensus on the information that should be extracted. Once this has been done, the data will be extracted in accordance with the determined criteria, and the results will be stored in a table. The key information that will be collected includes the following:

1. Author(s).
2. Year of publication.
3. Country of origin.
4. Aim of study.
5. Study design.
6. Population.
7. How was schizophrenia diagnosis determined?
8. How was ADHD diagnosis determined?
9. Psychostimulant: name, route of administration, dose, frequency and duration.
10. Determine if the patient was treated with antipsychotics: if so, name, route of administration, dose, frequency and duration.
11. The details of both Primary and secondary outcomes .

### Stage 5: collating, summarising and reporting the results

The objective of our scoping review is to present a narrative overview of the eligible texts. A descriptive summary of included studies will be presented which will include the study design, year the study was completed, the size of the population, location of the study and the primary outcome. A summary of the studies will be provided according to broad categories, such as the type of psychostimulant.

## Ethics and dissemination

The scoping review will use already publicly available empirical research; therefore, this study does not require ethical approval. As previously detailed, clinicians lack good quality evidence to help them make decisions relating to the prescribing of psychostimulants among patients with comorbid schizophrenia and ADHD. To our knowledge, this will be the first review to systematically examine the evidence to address this clinical scenario. This review, therefore, could help contribute to decision-making processes for this patient group, improving patient outcomes. Furthermore, as this review is designed to identify research gaps, we aim to contribute to the development of a research agenda that will benefit patients, clinicians and healthcare systems. Our dissemination strategy will involve open access peer review publication and scientific presentations.
