# Supplementary material for: Comorbid ADHD and schizophrenia and the use of psychostimulants: a scoping review protocol
Source: BMJ Open. 2024 Oct 23;14(10):e090290. doi: 10.1136/bmjopen-2024-090290 (PMC11499803; doi:10.1136/bmjopen-2024-090290)
Supplement: Supplementary file 1 [file bmjopen-2024-090290.reviewer_comments.pdf]

PEER REVIEW HISTORY

BMJ Open publishes all reviews undertaken for accepted manuscripts. Reviewers are asked to complete a checklist review form and are provided with free text boxes to elaborate on their assessment. These free text comments are reproduced below.

ARTICLE DETAILS

Title (Provisional)

Comorbid ADHD and schizophrenia, and the use of psychostimulants: a scoping review protocol

Authors

Bamford, Jordan; Qurashi, I; Axiaq, Ariana; Marwaha, Steven; Husain, Nusrat

---

VERSION 1 - REVIEW

**Reviewer**	**1**
**Name**	**Liu, Zongming**
**Affiliation**	**The Fifth People's Hospital of Nanning**
**Date**	**19-Jul-2024**
**COI**	**The authors declare that they have no conflict of interest**

IN Page 6 of 13 the author mentioned that ： with the focus on those published from

the year 20000 onwards。 I think there are some errors in this article， This article needs to be carefully revised

---

**Reviewer**	**2**
**Name**	**Ipsiroglu, Osman**
**Affiliation**	**The University of British Columbia, Pediatrics, Sleep/Wake-Behaviours**
**Date**	**24-Jul-2024**
**COI**	**None - this has been a mentored review, Dr. Scout McWilliams (https://orcid.org/0000-0003-1141-2775; BCCH-Research Institute, UBC and Sick Kids Hospital, UT) and I conducted the review.**

Thank you for allowing us to review the manuscript entitled, “Comorbid ADHD and schizophrenia, and the use of psychostimulants: a scoping review protocol” by Bamford et al. This protocol outlines the rationale and methods for a scoping review to investigate existing evidence for the use of psychostimulants in patients with comorbid ADHD and

schizophrenia. This is an exciting topic that will serve as an important review of the literature for future reference, as the effects of medications are often discussed in a controversial way. The protocol is well written but will benefit from some major changes prior to publication, as some of the core schizophrenia and ADHD symptoms are confounded by psychostimulant medication effects and worsening of core symptoms, e.g., sleep as a major contributing factor to both disorders. Further, the manuscript would also profit from a review of grammar and spelling.

Abstract

The abstract, particularly the introduction and 'Ethics & Dissemination' parts are well written and makes it transparent why this scoping review is conducted. The 'Methods & Analysis' part is also well written but might change after the suggested revisions of the protocol.

Strengths and Limitations

After revising the protocol, the authors may decide to expand limitations and reduce (or not) the planned workload, as the suggested inclusion and review of outcome measures in the studies eligible for analysis will drive the question how in-depth the analysis should be.

Introduction

The introduction is well written and provides an overview of the main schizophrenia and ADHD symptoms. The paragraph on schizophrenia ends with a short overview of the etiology. The second paragraph ends similar with an etiological concept ("dysfunction of the fronto-subcortical region of the brain, which is rich in dopamine") but the references are "old" and newer references since 2012 have investigated this further and suggest focusing on the iron deficiency pathway and pathways reviewing similarities in ADHD (Cortese et al. 2008; Silvestri 2022; Walters et al. 2008; McWilliams et al. 2022) and restlessness associated sleep disorders (DelRosso, Bruni, and Ferri 2018; Leung et al. 2020; Silvestri 2020; DelRosso et al. 2023).

Comorbidity, shared genetics and commonalities

In the context of schizophrenia, ADHD and associated sleep disturbances/disorders, many of the symptoms might be explained by sleep deprivation due to psychostimulant medication, and can be a cause of restless-ADHD like behaviors, (hypnagogic) hallucinations etc. The third paragraph proves this perspective, as all the listed symptoms are also the symptoms of chronically sleep deprived individuals (Marten et al. 2023; Stein, Weiss, and Hlavaty 2012).

Methods and analysis

Organized and have followed current guidelines for conducting a scoping review utilizing the framework outlined by Arksey & O'Malley, 2005. The following changes are suggested:

- The authors state that the aim of the scoping review is to “examine the use of psychostimulants in adults with ADHD and schizophrenia, irrespective of ADHD subtypes, comorbidities or medications (including antipsychotics)”. This statement, however, seems to contradict one of the research questions which is specifically looking at patients who are also prescribed antipsychotics (which also affect sleep, due to effects on the sleep architecture).

- The authors state that they will “not restrict by age”, however in the first sentence of this paragraph, it is stated that the population of interest is “adults over the age of 18”. This point needs to be clarified.

- In the section “Stage 2: identification of relevant studies”, the second paragraph starting with “the population in this scoping review”, would be better suited in the next section “Stage 3: Study Selection” as some of this information is redundant.

- Was the search strategy developed with the help of a librarian? If so, this should be stated.

- Year “20000” should be “2000”.

- On review of the search strategy outlined in Table 2, there appear to be a relatively small number of studies that come up. Therefore, the authors may want to re-think their inclusion and exclusion criteria, mainly restricting to studies published after 2000 as well as limiting to populations of age 18 and up. As the majority of individuals are diagnosed with ADHD in childhood, this is often when psychostimulant medications would be started. Therefore, possibly even expanding the scope of this review, to include patients, not only with a formal diagnosis of schizophrenia, but also those presenting with psychosis or other symptoms indicative of schizophrenia may be a good way to ensure you are capturing all of the existing literature.

- “Study 3: Study selection” should read “Stage 3: Study selection”

- The authors should include more details about the inclusion and exclusion criteria. At present, they have only included a section on “eligibility requirements” however this section is missing key aspects such as study type (they mentioned above in the previous section that they would not restrict by study design, but this should be included within the inclusion criteria). Similarly, the authors need to specify more about the population they are looking at (currently only referring to the diagnosis, but also need to include age – this is stated in the section above, but again, would be more appropriate in the “Stage 3: Study selection” section where all of this information should be clearly outlined).

- In “Stage 4: Charting the data”, the authors should include more details about the outcomes that they will be assessing, this includes: type of outcome measure (e.g., primary vs secondary vs treatment emergent adverse events. E.g., will the authors only be looking at primary outcome measures or others as well?).

- The authors should specify what information they will be collecting about the “form of psychostimulant used”. Information about drug name/class, route of administration, dose

and frequency of administration, medication timing, and duration of use would be important information to collect. Similarly with antipsychotics, the authors state “were patient on antipsychotics”; is this simply a yes/no question, or will additional information be collected? We would highly recommend that information collected about any medication be comprehensive as we have suggested above.

- In “Stage 5: Collating, summarising and reporting results” the authors state that a quantitative assessment will not be employed. However, it would be important for the authors to, at a minimum, carry out descriptive statistics of study characteristics such as population, study design, outcomes, etc.

Cortese, Samuele, Michel Lecendreux, Bernardo Dalla Bernardina, Marie Christine Mouren, Andrea Sbarbati, and Eric Konofal. 2008. “Attention-Deficit/hyperactivity Disorder, Tourette’s Syndrome, and Restless Legs Syndrome: The Iron Hypothesis.” Medical Hypotheses 70 (6): 1128–32.

DelRosso, Lourdes M., Oliviero Bruni, and Raffaele Ferri. 2018. “Restless Sleep Disorder in Children: A Pilot Study on a Tentative New Diagnostic Category.” Sleep 41 (8). https://doi.org/10.1093/sleep/zsy102.

DelRosso, Lourdes M., Maria Paola Mogavero, Oliviero Bruni, and Raffaele Ferri. 2023. “Restless Legs Syndrome and Restless Sleep Disorder in Children.” Sleep Medicine Clinics 18 (2): 201–12.

Leung, Wayne, Ishmeet Singh, Scout McWilliams, Sylvia Stockler, and Osman S. Ipsiroglu. 2020. “Iron Deficiency and Sleep – A Scoping Review.” Sleep Medicine Reviews. https://doi.org/10.1016/j.smrv.2020.101274.

Marten, Finja, Lena Keuppens, Dieter Baeyens, Bianca E. Boyer, Marina Danckaerts, Samuele Cortese, and Saskia Van der Oord. 2023. “Sleep Parameters and Problems in Adolescents with and without ADHD: A Systematic Review and Meta-Analysis.” JCPP Advances 3 (3): e12151.

McWilliams, Scout, Ishmeet Singh, Wayne Leung, Sylvia Stockler, and Osman S. Ipsiroglu. 2022. “Iron Deficiency and Common Neurodevelopmental Disorders-A Scoping Review.” PloS One 17 (9): e0273819.

Silvestri, Rosalia. 2020. “The importance of diagnosing and treating iron deficiency in sleep disorders .” Sleep Medicine Reviews. England: Elsevier Ltd. https://doi.org/10.1016/j.smrv.2020.101314.

———. 2022. “Sleep and ADHD: A Complex and Bidirectional Relationship.” Sleep Medicine Reviews.

Stein, Mark A., Margaret Weiss, and Laura Hlavaty. 2012. “ADHD Treatments, Sleep, and Sleep Problems: Complex Associations.” Neurotherapeutics: The Journal of the American Society for Experimental NeuroTherapeutics 9 (3): 509–17.

Walters, Arthur S., Rosalia Silvestri, Marco Zucconi, Ranju Chandrashekariah, and Eric Konofal. 2008. “Review of the Possible Relationship and Hypothetical Links between Attention Deficit Hyperactivity Disorder (ADHD) and the Simple Sleep Related Movement Disorders, Parasomnias, Hypersomnias, and Circadian Rhythm Disorders.” Journal of Clinical Sleep Medicine: JCSM: Official Publication of the American Academy of Sleep Medicine 4 (6): 591–600.

VERSION 1 - AUTHOR RESPONSE

**Reviewer 1**	
Point raised	Action
IN Page 6 of 13 the author mentioned that ：with the focus on those published from the year 20000 onwards。I think there are some errors in this article， This article needs to be carefully revised	Thank you for this point. We have amended the manuscript, it now reads 2000 rather than 20000. We have thoroughly reviewed the manuscript for grammar and spelling and have made numerous amendments– these can be reviewed on the tracked changes version.
Reviewer 2	
The abstract, particularly the introduction and ‘Ethics & Dissemination’ parts are well written and makes it transparent why this scoping review is conducted. The ‘Methods & Analysis’ part is also well written but might change after the suggested revisions of the protocol.	Thank you – we have updated the methods and analysis with the following points and feedback – we appreciate your input and believe the protocol has been strengthened by your input.
After revising the protocol, the authors may decide to expand limitations and reduce (or not) the planned workload, as the suggested inclusion and review of outcome measures in the studies eligible for analysis will drive the question how in-depth the analysis should be	Thank you – we have elected to retain the current strengths and limitations.<br><br>However, your points raised, specifically about the role of sleep disturbance as a commonality between ADHD and schizophrenia, and the overlapping symptoms, will be reflected in our discussion and limitations of the completed scoping review.
The introduction is well written and provides an overview of the main schizophrenia and ADHD symptoms. The paragraph on schizophrenia ends with a short overview of the etiology. The second paragraph ends similar with an etiological concept (“dysfunction of the fronto-subcortical region of the brain, which is rich in	Thank you for raising this important and interesting point.<br><br>We have now integrated some of the recommended texts, specifically:<br><br>McWilliams S, Singh I, Leung W, Stockler S, Ipsiroglu OS. Iron deficiency and common

dopamine") but the references are "old" and newer references since 2012 have investigated this further and suggest focusing on the iron deficiency pathway and pathways reviewing similarities in ADHD (Cortese et al. 2008; Silvestri 2022; Walters et al. 2008; McWilliams et al. 2022) and restlessness associated sleep disorders (DelRosso, Bruni, and Ferri 2018; Leung et al. 2020; Silvestri 2020; DelRosso et al. 2023).	neurodevelopmental disorders-A scoping review. PLoS One. 2022 Sep 29;17(9):e0273819. doi: 10.1371/journal.pone.0273819. PMID: 36173945; PMCID: PMC9522276<br><br>Marten, F., Keuppens, L., Baeyens, D., Boyer, B. E., Danckaerts, M., Cortese, S., & Van der Oord, S. (2023). Sleep parameters and problems in adolescents with and without ADHD: A systematic review and meta-analysis. JCPP advances, 3(3), e12151.<br><br>We have also added more up to date references such as:<br><br>Bernanke, J., Luna, A., Chang, L., Bruno, E., Dworkin, J., & Posner, J. (2022). Structural brain measures among children with and without ADHD in the Adolescent Brain and Cognitive Development Study cohort: a cross-sectional US population-based study. The Lancet Psychiatry, 9(3), 222-231.
In the context of schizophrenia, ADHD and associated sleep disturbances/disorders, many of the symptoms might be explained by sleep deprivation due to psychostimulant medication, and can be a cause of restless-ADHD like behaviors, (hypnagogic) hallucinations etc. The third paragraph proves this perspective, as all the listed symptoms are also the symptoms of chronically sleep deprived individuals (Marten et al. 2023; Stein, Weiss, and Hlavaty 2012).	Thank you for this interesting contribution.<br><br>We have integrated the link between schizophrenia, ADHD and sleep – in the introduction it states 'Those with ADHD compared to healthy controls have significant disrupted sleep'<br><br>In the comorbidity, shared genetics and commonalities we state 'Many of the common symptoms are also in part possibly explained by underlying sleep disturbance which is common in both conditions (Carruthers et al., 2021; Marten et al., 2023).'<br><br>Importantly, we would reflect on this point in the discussion of our completed scoping review.
The authors state that the aim of the scoping review is to "examine the use of psychostimulants in adults with ADHD and schizophrenia, irrespective of ADHD subtypes, comorbidities or medications (including antipsychotics)". This statement, however, seems to contradict one of the research questions which is specifically looking at patients who are also prescribed antipsychotics	Thank you for this important point – we have now removed this statement to improve clarity.

(which also affect sleep, due to effects on the sleep architecture).	
The authors state that they will "not restrict by age", however in the first sentence of this paragraph, it is stated that the population of interest is "adults over the age of 18". This point needs to be clarified.	Thank you for this point, we have updated the text for clarity.
In the section "Stage 2: identification of relevant studies", the second paragraph starting with "the population in this scoping review", would be better suited in the next section "Stage 3: Study Selection" as some of this information is redundant.	Thank you for this point, we have restructured this section in line with your feedback.
Was the search strategy developed with the help of a librarian? If so, this should be stated.	Thank you – the search strategy was not developed with a librarian, rather experts in the field with previous experience and training in scoping reviews.
Year "20000" should be "2000".	Thank you – we have corrected this.
On review of the search strategy outlined in Table 2, there appear to be a relatively small number of studies that come up. Therefore, the authors may want to re-think their inclusion and exclusion criteria, mainly restricting to studies published after 2000 as well as limiting to populations of age 18 and up. As the majority of individuals are diagnosed with ADHD in childhood, this is often when psychostimulant medications would be started. Therefore, possibly even expanding the scope of this review, to include patients, not only with a formal diagnosis of schizophrenia, but also those presenting with psychosis or other symptoms indicative of schizophrenia may be a good way to ensure you are capturing all of the existing literature.	Thank you for this point. The points raised are interesting and useful – but we wish to point out that we see the specific scope of this review to focus on those over 18, with a diagnosis of ADHD and schizophrenia. While we may restrict the number of studies which could be included, the aim of scoping review is also to explore where further research is needed, this will still be beneficial.<br>The focus on these parameters is to make the findings relevant for clinicians and prescribers, at present those working in general adult psychiatry settings working with patients with this comorbidity lack good quality guidelines.
"Study 3: Study selection" should read "Stage 3: Study selection"	Thank you – we have corrected this.
The authors should include more details about the inclusion and exclusion criteria. At present, they have only included a section on "eligibility requirements" however this section is missing key aspects such as study type (they mentioned above in the previous section that they would not restrict by study design, but this should be included within the inclusion criteria). Similarly, the authors need to specify more about the population they are looking at (currently only referring to the diagnosis, but also need to include age – this is stated in the section above, but again, would be more appropriate in the	Thank you.<br>In the eligibility requirements for title/abstract we have now included the age parameter and any study design.

"Stage 3: Study selection" section where all of this information should be clearly outlined).	
In "Stage 4: Charting the data", the authors should include more details about the outcomes that they will be assessing, this includes: type of outcome measure (e.g., primary vs secondary vs treatment emergent adverse events. E.g., will the authors only be looking at primary outcome measures or others as well?).	Thanks you for this important point. We have now specified that we will explore both primary and secondary outcomes.<br>We do highlight that the charting of data will be an iterative process – we will use our small sample of studies initially to gain consensus on what exactly we will collect.
The authors should specify what information they will be collecting about the "form of psychostimulant used". Information about drug name/class, route of administration, dose and frequency of administration, medication timing, and duration of use would be important information to collect. Similarly with antipsychotics, the authors state "were patient on antipsychotics"; is this simply a yes/no question, or will additional information be collected? We would highly recommend that information collected about any medication be comprehensive as we have suggested above.	Thank you for this important point.<br>This section now reads:<br>9. Psychostimulant: Name, route of administration, dose, frequency and duration<br>10. Determine if patient was treated with antipsychotics: if so name, route of administration, dose, frequency and duration<br><br>We do highlight that the charting of data will be an iterative process – we will use our small sample of studies initially to gain consensus on what exactly we will collect.
In "Stage 5: Collating, summarising and reporting results" the authors state that a quantitative assessment will not be employed. However, it would be important for the authors to, at a minimum, carry out descriptive statistics of study characteristics such as population, study design, outcomes, etc.	Thank you. This section now reads 'The objective of our scoping review is to present a narrative overview of the eligible texts. A descriptive summary of included studies will be presented which will include the study design, year the study was completed, the size of the population, location of the study and the primary outcome. A summary of the studies will be provided according to broad categories, such as the type of psychostimulant. The results will be presented in tabular form and will be accompanied by a description of their applicability to the specific research questions.'

VERSION 2 - REVIEW

Reviewer 2

Name Ipsiroglu, Osman

Affiliation The University of British Columbia, Pediatrics, Sleep/Wake-Behaviours

Date 27-Sep-2024

COI

The authors have addressed all reviewer comments satisfactorily. This protocol is now suitable for publication. Thank you for making these necessary changes and/or adding an explanation.
